# Neutrophil Interactions Stimulate Evasive Hyphal Branching by *Aspergillus fumigatus*

**DOI:** 10.1371/journal.ppat.1006154

**Published:** 2017-01-11

**Authors:** Felix Ellett, Julianne Jorgensen, Galit H. Frydman, Caroline N. Jones, Daniel Irimia

**Affiliations:** 1 BioMEMS Resource Center, Division of Surgery, Innovation and Bioengineering, Department of Surgery, Massachusetts General Hospital, Shriners Burns Hospital, Harvard Medical School, Massachusetts, United States of America; 2 Division of Comparative Medicine, Department of Biological Engineering, Massachusetts Institute of Technology, Cambridge, Massachusetts, United States of America; Texas A&M University, UNITED STATES

## Abstract

Invasive aspergillosis (IA), primarily caused by *Aspergillus fumigatus*, is an opportunistic fungal infection predominantly affecting immunocompromised and neutropenic patients that is difficult to treat and results in high mortality. Investigations of neutrophil-hypha interaction *in vitro* and in animal models of IA are limited by lack of temporal and spatial control over interactions. This study presents a new approach for studying neutrophil-hypha interaction at single cell resolution over time, which revealed an evasive fungal behavior triggered by interaction with neutrophils: Interacting hyphae performed *de novo* tip formation to generate new hyphal branches, allowing the fungi to avoid the interaction point and continue invasive growth. Induction of this mechanism was independent of neutrophil NADPH oxidase activity and neutrophil extracellular trap (NET) formation, but could be phenocopied by iron chelation and mechanical or physiological stalling of hyphal tip extension. The consequence of branch induction upon interaction outcome depends on the number and activity of neutrophils available: In the presence of sufficient neutrophils branching makes hyphae more vulnerable to destruction, while in the presence of limited neutrophils the interaction increases the number of hyphal tips, potentially making the infection more aggressive. This has direct implications for infections in neutrophil-deficient patients and opens new avenues for treatments targeting fungal branching.

## Author Summary

For patients who have compromised immune systems, infection with yeasts and moulds are often deadly. To learn more about how immune cells fight fungal infection, we developed an “infection-on-a-chip” device, which allowed us to directly image interactions in real time. To our surprise, when immune cells attacked the fungi and stalled its growth, the fungi formed new branches, allowing it to avoid the interaction and continue growing. Our hypothesis is that in people with healthy immune cells, forcing the fungi to branch may make it more vulnerable to further attack. However, in patients with impaired immune systems, increasing the number of fungal branches may make the infection more aggressive. By developing new drugs that affect fungal branching, we may be able to give the immune cells of sick patients an advantage in their uphill battle against fungal infection.

## Introduction

*Aspergillus fumigatus* is a filamentous, conidiating fungus ubiquitous to the environment. Airborne conidia are inhaled in the hundreds by individuals every day [1] and are quickly cleared by macrophages [2–4]. If not cleared, conidia grow into elongated hyphal structures, which present a unique challenge for the cellular immune system: Hyphae cannot be phagocytosed by immune cells, requiring deployment of extracellular antifungal mechanisms such as degranulation [5], production of extracellular reactive oxygen species (ROS) [6], secretion of ion-sequestering proteins such as Calprotectin [7] and Lactoferrin [8], and production of neutrophil extracellular traps (NETs) [9–11]. However, the fungus often overcomes these immune mechanisms and infections (aspergillosis) are frequent in particular immunosuppressed patients [12] and those suffering from chronic granulomatous disease (CGD) [13] or chronic obstructive pulmonary disease (COPD) [14]. The mechanisms by which *Aspergillus* can avoid both cellular and extracellular antifungal mechanisms are not well understood, hampering our ability to develop effective treatments to avoid aspergillosis in these patients [15].

The presence of dichotomously branched hyphae has long been used as a diagnostic indicator for invasive *A*. *fumigatus* infection [16]. It has been generally assumed that controlled branching enhances fungal invasion, as strains that exhibit perturbed branching behavior and irregular growth polarity exhibit reduced virulence *in vivo* [17–19]. Perturbed branching in these mutant strains is driven by altered ROS production or localization [20,21], calcium signalling [18,22], hypoxic stress responses [19], or cell wall synthesis pathways [23,24]. However, the study of host-hyphae interactions *in vitro* and in animal models of IA is limited by lack of control over the interactions and lack of methods to study these interactions at single cell resolution.

Microscale technologies, particularly microfluidic devices, are emerging as an important tool for *ex vivo* analysis of neutrophil biology [25]. Recent work has begun to explore the applicability of these devices to investigating host-pathogen interaction [26,27]. For example, work by our group recently demonstrated that migration along chemotactic gradients acts to prime neutrophils for antifungal activity against *A*. *fumigatus* [27], while others have used microfluidic techniques to identify immunosuppressive fungal metabolites [28]. For microbiologists, microfluidics has provided a useful platform for imaging active *Candida* hyphal growth at high resolution, revealing that internal polarity protein complexes are asymmetrically skewed toward softer substratum [29].

Here we present an *in vitro* microfluidic assay that enables us to study interactions between neutrophils and hyphae at cellular resolution over time. We designed microfluidic devices to confine the growing hyphae in linear channels, enabling high spatial and temporal resolution monitoring of growing hyphal tips. These new microfluidic devices facilitate live study of interactions in unprecedented detail. Key features of the microfluidic device include spatial control of hypha growth, allowing alignment of multiple hyphae in parallel lanes, as well as temporal and spatial control of interactions between hyphae and neutrophils, facilitating detailed imaging studies. Using the new tools, we systematically explored the mechanisms by which neutrophils trigger hyphal branching. Furthermore, we designed microfluidic devices to decouple branching from neutrophil interaction, using mechanical obstacles. This enabled us to quantify and compare the effect of neutrophils on hyphae at different stages of branching, from thick, primary hyphae through to thin, highly branched structures.

We find that *A*. *fumigatus* branching after interaction with human neutrophils is independent of neutrophil NADPH oxidase activity and NETosis, but can be phenocopied by iron chelation and mechanical or physiological stalling of hyphal tip extension. Evasive branching and the consequential increased number of hyphal tips can enable the fungus to continue growing aggressively in the presence of small numbers of neutrophils. However, in the presence of sufficient numbers of neutrophils, branching increases fungus vulnerability to neutrophil destruction. Our findings suggest that new approaches to controlling *A*. *fumigatus* infections in patients are possible, and provide a new set of tools to further explore these possibilities for drug discovery.

## Results

### *De novo* hyphal branching is stimulated by neutrophil-hypha interactions

**Fig 1:**
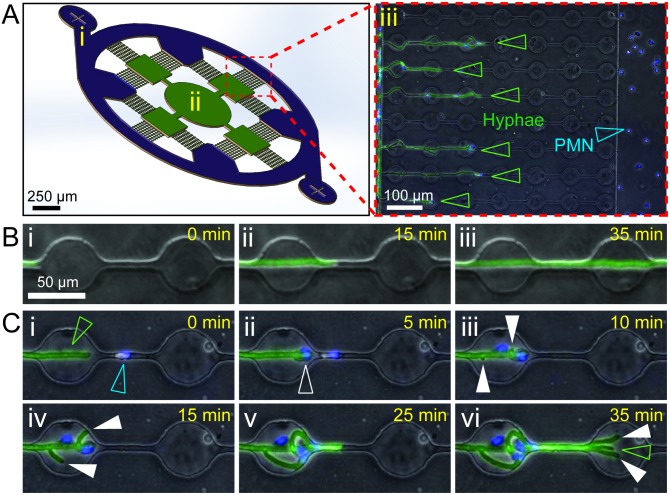
Neutrophil interactions induce hyphal branching. (A) Schematic of microfluidic device used in the study. The device consists of a circular outer channel and neutrophil loading ports (i, purple), inner conidia loading port (ii) and hyphal growth chambers (green), and 12 viewing fields consisting of 9 conidia-hypha interaction channels per field (magnified in iii). (iii) The interaction channels guide parallel growth of individual hypha (EGFP, open green arrowheads) from the hyphal growth chambers and enables observation of interactions with neutrophils/polymorphonuclear (PMN) leukocytes (Hoechst, open blue arrowheads) loaded into the circular outer channel (S1 Movie). (B) Hyphal growth through the interaction channels in the absence of neutrophils is linear and rarely involves spontaneous branching (<5%). (C) Rapid induction of hypha branching upon interaction with neutrophils (S2 Movie). (i) Neutrophils (Hoechst, open blue arrowhead) are recruited to the hyphal tip (EGFP, open green arrowhead) and make contact with the hypha (ii, open white arrowhead). This rapidly induces formation of new hyphal tips (iii-iv, full white arrowheads) proximal to the interaction. (vi) This may result in amplification of hypha number, with new hypha (full white arrowheads) adding to existing hypha tips (open green arrowhead).

To allow measurement of reproducible and controllable leukocyte-hypha interactions, we designed a microfluidic device to confine the growing hyphae along a single axis. The device consists of parallel channels with circular “swarming chambers” separating a fungal loading chamber from a neutrophil loading chamber (Fig 1A). The device is multiplexed to allow 12 fields of view per device. Moreover, the use of microfluidic devices for microscopy enabled high throughput and high-resolution imaging in a single plane (Fig 1A). Each viewing field accommodates 9 parallel interaction channels, allowing the potential for simultaneous capture of over 100 individual, interacting hyphae per condition, with 12 conditions tested per experiment. Conidia were seeded into the central chamber of the device (Fig 1Aii) and hyphae cultured for 18 hours, until they began entering the interaction channels (Fig 1Aiii). Hypha entered 4–5 of the 9 interaction channels on average (S1A Fig), with a slight bias observed toward the far viewing field, as might be expected for hyphae exhibiting primarily radial growth (S1A Fig). Chemical treatments could be administered throughout the entire device by loading through the central chamber, or selectively to the growing tips of the hypha by administering into the outer channel via the neutrophil loading port (Fig 1Ai).

**Fig 2:**
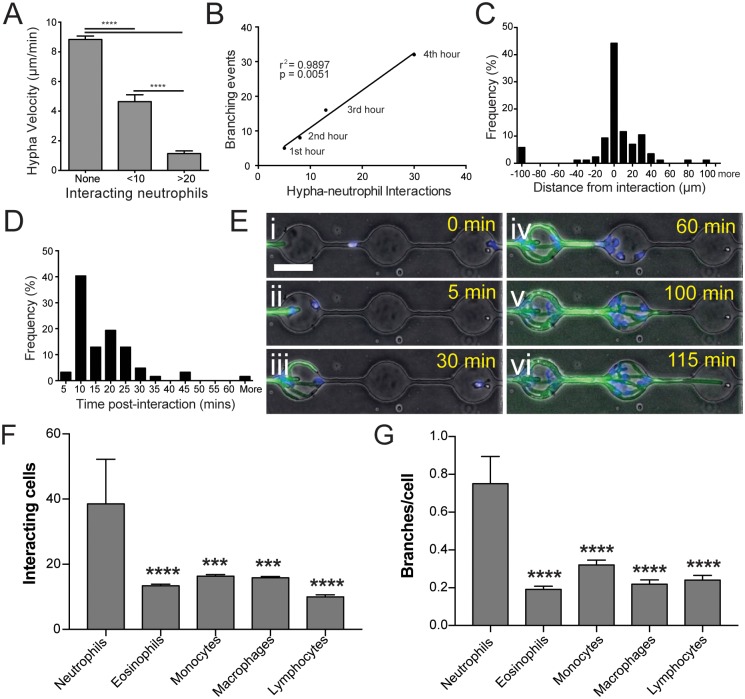
Interaction with neutrophils induces hyphal branching. (A) Amplitude of neutrophil response correlates to reduction in velocity of hyphal growth, with larger numbers of neutrophils better able to slow growth. N ≥ 10 measurements from n ≥ 5 representative hyphae per group. (B) Induction of branching over time shows a linear correlation with number of neutrophil interactions. (C) Induction of new branches is enriched proximally to neutrophil interactions, with most branches occurring within 20–30 μm of an interaction point. (D) Following interaction, most branches are induced within 5–25 minutes post interaction. (E) Robust recruitment of neutrophils amplifies branch induction (iii-v, S2 Movie) and slows hyphal growth. However, at least one hypha branch is usually able to avoid confrontation and continue growing unobstructed (vi). (F) Neutrophils are recruited to fungal hyphae in significantly higher numbers than other immune cells. (G) Neutrophil interaction with hyphae induces apical branching at more than twice the rate of other immune cells. N ≥ 24 hyphae scored from N ≥ 4 experiments. Error bars: mean ± SEM. Statistics: one-way ANOVA with Tukey’s post test. Adjusted p values: *p≤0.05, **p≤0.01, ***p≤0.001, ****p≤0.0001.

Delivery of neutrophils into the outer channel was followed by migration down the interaction channels towards the growing hyphae (S1 Movie). Upon interaction of neutrophils with the hyphae, particularly when close to the growing tip, we observed an unexpected hyphal response: The hyphae formed new growth tips and branched, a phenotype we hypothesized to represent damage-induced “evasive branching” (Fig 1Ciii–1Civ, S2 Movie). Neutrophils engaged new hyphal tips and inhibited their growth, resulting in a transient decrease in leading tip velocity (Fig 2A). However, at least one hypha was almost always observed to avoid confrontation and continue penetrative growth (Fig 2Evi, S2 Movie).

The frequency of hyphal branching was proportional to the number of interacting neutrophils (Fig 2B), occurred proximally to sites of interaction (Fig 2C), and began shortly following initiation of the interaction (Fig 2D). During induction of apical branching, neutrophil interaction decreased the growth velocity of the leading hyphal tip, relative to the number of neutrophils involved (Fig 2A and 2E). To test the specificity of branch induction to neutrophil interaction, we repeated this assay using a range of other primary human immune cells: eosinophils; monocytes; monocyte-derived macrophages, and lymphocytes (Fig 2F and 2G). Although comparable numbers of each cell type were delivered, fewer interactions with hyphae were observed for these cells compared to neutrophils, as they exhibited little recruitment to fungal hyphae (Fig 2F). When they were observed to interact, apical branch induction occurred at less than half the rate for interaction with these cell types compared to neutrophils (Fig 2G), while induction of lateral branching was not observed at all. Together, these correlative observations strongly support a model in which hyphae form new branches in response to neutrophil interactions.

### Induction of apical hyphal branching is independent of neutrophil NADPH activity and NETosis

**Fig 3:**
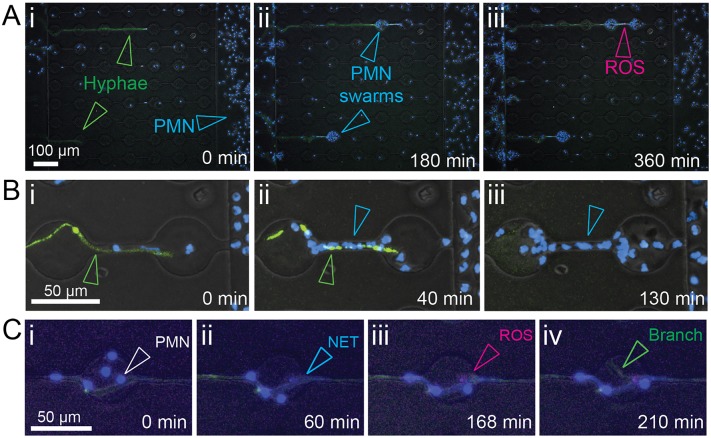
Neutrophils display antifungal activities. (A) Imaging of hyphae and neutrophil behavior within interaction channels (i) demonstrates robust recruitment of neutrophils (Hoechst, open blue arrowheads) to EGFP-expressing fungi (open green arrowheads) and formation of swarms (ii, open blue arrowheads) while production of reactive oxygen species (iii, ROS, open magenta arrowhead) can be visualized by staining with CellROX. (B) Killing of hyphae by neutrophils can be visualised by quenching of cytoplasmic GFP fluorescence following breach of the hyphal cell wall. (C) Neutrophils attach to the hyphae (i) and undergo NETosis (ii). Production of ROS (iii) and formation of new lateral branches (iv) can be observed at sites of neutrophil interaction.

Following delivery of neutrophils, robust antifungal responses were observed, including attachment of neutrophils both to the growing tip and along the length of hypha (Fig 3), formation of neutrophil swarms (Fig 3Aii), NETosis (Fig 3Cii) and generation of ROS (Fig 3Aiii and 3Ciii).

Given that neutrophil interaction induced formation of new hyphal tips, we hypothesized that this induction is dependent on neutrophil antimicrobial mechanisms. To test this hypothesis, we performed a series of experiments aimed at inhibiting or enhancing specific neutrophil functions.

**Fig 4:**
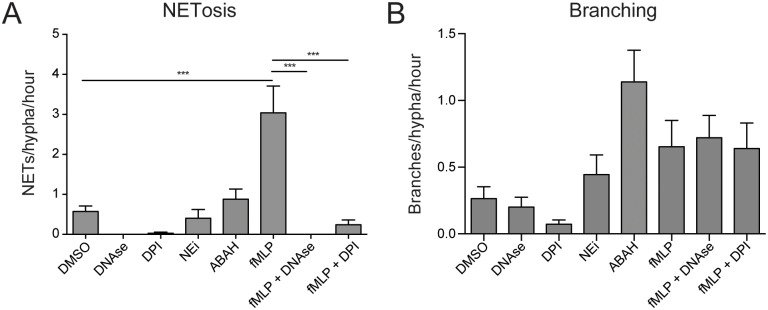
Apical branching is induced at sites of neutrophil interaction independently of ROS production and NETosis. (A) Treatment with known regulators of NETosis allowed manipulation of NET formation by neutrophils in this system. (B) Inhibition of NETosis by DPI or DNAse treatment had no influence on the increased branching levels observed in fMLP-treated groups, suggesting that this effect was independent of NETosis. N ≥ 25 hyphae scored from 3 experiments. Errors bars: mean ± SEM. Statistics: One-way ANOVA with Tukey’s post test. Adjusted p values: *p≤0.05, **p≤0.01, ***p≤0.001, ****p≤0.0001.

Our previous studies demonstrated that neutrophils primed by an N-formyl-methionine-leucyl-phenylalanine (fMLP) gradient exhibited enhanced suppression of *A*. *fumigatus* growth [27]. Introduction of an fMLP gradient in the current study prior to delivery of neutrophils resulted in increased recruitment of neutrophils to sites of interaction (S1B Fig), an increase in hyphal exposure to NETs (Fig 4A), and increased fungal branching (Fig 4B). We therefore hypothesized that NETosis might be central to inducing hyphal branching.

Partial inhibition of NETosis has been demonstrated by reducing the activity of Neutrophil Elastase or Myeloperoxidase by pre-treatment Neutrophil Elastase inhibitor (NEi) or 4-aminobenzoic acid hydrazide (ABAH) respectively [8,30]. Pre-treatment of neutrophils with these chemicals did not significantly reduce NETosis or effect branching in this system (Fig 4A and 4B). Inhibition of NADPH oxidase using diphenyleneiodonium chloride (DPI) successfully abrogated the enhanced NETosis observed for fMLP-primed neutrophils (Fig 4A), but reducing NETosis in this manner appeared to have no effect on hyphal branch induction (Fig 4B). Degrading NETs using DNAse, one of the most robust methods of reducing NETs from healthy neutrophils [31,32], also failed to effect hyphal branch induction (Fig 4B), suggesting that branch induction occurs independently on NETosis.

### Hyphal “hyperbranching” is induced by heat-labile plasma components

**Fig 5:**
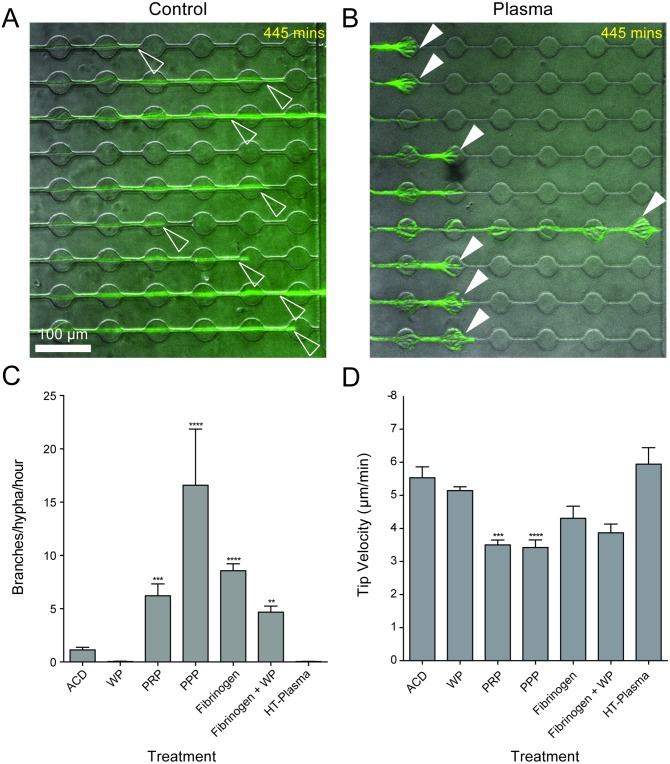
Hyperbranching is induced by heat-labile plasma factors independently of complement, platelets, and clot formation. (A) Representative image of hyphal growth (green, EGFP, open white arrowheads) in the absence of treatment (B) Representative image of plasma-induced hyperbranching (green, EGFP, filled white arrowheads) (C) Plasma preparations, but not washed platelets, induce hyphal hyperbranching. This effect could be phenocopied by addition of Fibrinogen and abrogated by heat-treatment. (D) Hyphal velocity was significantly slower during growth toward platelet rich plasma and PPP, but was not effected by washed platelets (WP). N ≥ 18 hyphae scored per condition from 3 experiments. Error bars: mean ± SEM. Statistics: One-way ANOVA with Tukey’s post test. Adjusted p values: *p≤0.05, **p≤0.01, ***p≤0.001, ****p≤0.0001.

In a physiological setting, platelets have been demonstrated to coordinate with neutrophils to enhance antifungal activity [33]. To test whether this phenotype could be recapitulated in our system, autologous platelet poor plasma (PPP), platelet rich plasma (PRP), or washed platelets (WP) were added alone or immediately preceding neutrophil delivery. Strikingly, we observed robust stimulation of hyphal hyperbranching in response to exposure to PPP and PRP, but not WP, even in the absence of neutrophils (Fig 5A and 5B). Lack of hyperbranching in the acid citrate dextran (ACD)-containing media control and the WP group ruled out a role for ACD in induction of this response.

Induction of the hyperbranching response appeared to coincide with formation of cross-linked fibrin clots in the plasma groups (S3 Movie). To test whether clot formation was sufficient to induce hyphal branching, we spiked WP with fluorescently labelled Fibrinogen, which formed robust clots upon platelet activation by the hyphae. This treatment increased hyphal branching relative to the ACD control (Fig 5C). However, the non-clotting control treatment of Fibrinogen alone induced additional branching compared to the clotting treatment group, demonstrating that Fibrinogen alone is sufficient to induce branching and that formation of cross-linked fibrin clots was dispensable for this response (Fig 5C).

Plasma is a complex mix of proteins, peptides and various chemicals, some of which are stable at 56°C, such as Fibrinogen, and some of which are degraded, such as Complement factors. Treatment of hyphae with heat-treated (HT)-plasma did not induce hyperbranching (Fig 5C), ruling out endogenous Fibrinogen as the inducing factor present in plasma, and instead implicating a heat-labile component in this pathway.

Importantly, plasma-induced hyperbranching significantly attenuated leading tip velocity, relative to the number of branches produced (Fig 5C and 5D), suggesting a strong link between increased branching and decreased hyphal tip growth speed.

### Apical branching can be induced by mechanical impedance of hyphal tip extension

The ability of non-commensal fungi to sense and avoid physical obstacles (thigmotropism) plays an important role in their hyphal growth in their primary environment, and is also implicated in virulence [34]. Since induction of branching was observed following decreased hyphal growth speed in response to increased neutrophil recruitment (Fig 2A, S2 Movie), we hypothesized that hypha might be responding to mechanical impedance of leading tip growth.

**Fig 6:**
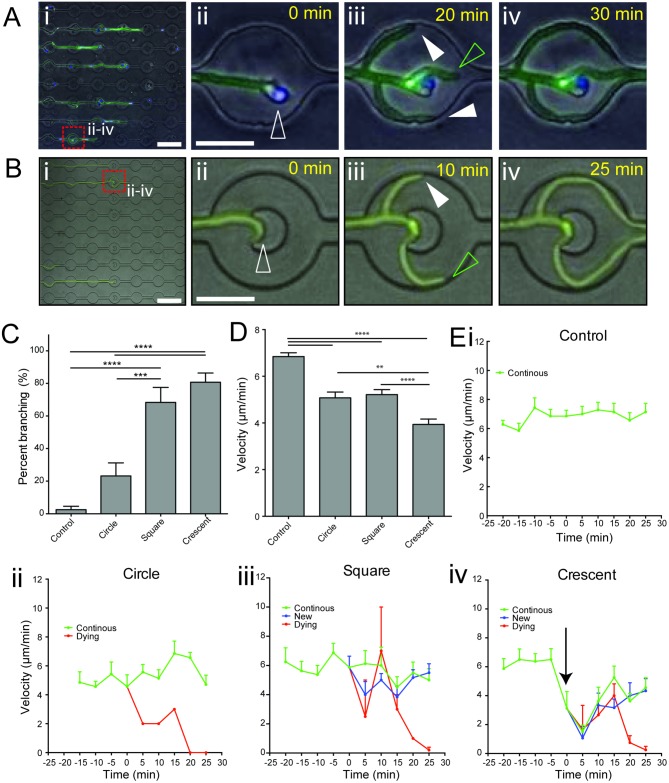
Mechanical impedance of tip extension induces apical hyphal branching. (A) Representative example of branch induction following interaction with a single neutrophil. (i) Hyphal growth (green) through channels and interactions with neutrophils (blue), as observed in time-lapse microscopy (S1 Movie) (ii-iv) Magnified view of frames extracted from time-lapse microscopy of interactions (dashed red square box in i), which demonstrate formation of actively growing new hypha tips (full white arrowheads) following interaction with a single neutrophil (open white arrowhead), in addition to the primary tip (open green arrowhead). (B) Induction of branching during interaction of the hyphal tip with a crescent-shaped obstacle. (i) Hyphal growth (green) through channels with obstacles, as observed in time-lapse microscopy (S4 Movie) (ii-iv) Magnified view of frames extracted from time-lapse microscopy (dashed red square box in i), show efficient formation of a new hypha tip (full white arrowhead) in addition to the primary tip (open green arrowhead). (C) Comparison of branch induction by differently shaped obstacles. Square and crescent shaped obstacles provided robust branch induction. (D) Comparison of average hyphal tip velocity during interaction period (-20 to +25 mins relative to contact with obstacle). Crescent-shaped obstacles slowed hyphal tip extension most efficiently during interaction. (E) Graphing of instantaneous hyphal tip velocity during interaction with differently shaped obstacles (i-iv). Crescent shaped obstacles induced a striking decrease in tip velocity during the interaction, just prior to branching (black arrow indicates steep drop in tip velocity). N ≥ 9 measurements per condition from n ≥ 7 representative hyphae per group. Errors bars: mean ± SEM. Statistics: One-way ANOVA with Tukey’s post test. Adjusted p values: *p≤0.05, **p≤0.01, ***p≤0.001, ****p≤0.0001.

To test this hypothesis, we developed a new device that incorporated a range of physical obstacles into our microfluidic channels, designed to apply variable mechanical impedance to the growing tip upon contact. During interaction with the hypha tip, these obstacles induced apical branching similar to that observed during neutrophil interaction (Fig 6A and 6B), at a frequency relative to the ability of the obstacle to slow tip extension (Fig 6C and 6D). A crescent-shaped “tip-trap” obstacle, which efficiently slowed tip extension (Fig 6D and 6E) induced branching at a rate of more than 80% (Fig 6B and 6C, S4 Movie), demonstrating that physical stalling of tip extension is sufficient to induce hyphal branching.

### Apical branching can be induced by thermally stalling hyphal tip extension

Since single neutrophils were observed to induce apical branching (Fig 6A) and seemed unlikely to be physically blocking hyphal extension, we hypothesized that they might instead induce branching by physiologically stalling tip extension.

**Fig 7:**
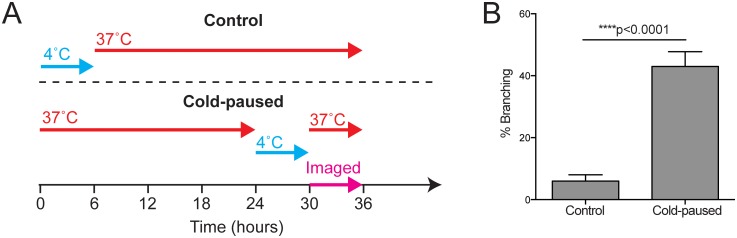
Pausing growth by temperature shifts induces apical branching. (A) Thermal inhibition of hyphal growth at 4°C followed by return to 37°C allowed the effect of pausing hyphal growth to be compared to continuously growing controls. (B) Cold-paused hyphae exhibited robust branching at hyphal tips upon return to growth conditions compared to continuously growing hyphae. N ≥ 178 hyphae per condition scored from 3 experiments. Errors bars: mean ± SEM. Statistics: Student’s T-test.

To test whether physiologically stalling growth was sufficient to induce apical branching, we compared branching rates between actively growing hyphae and hyphae whose growth had been arrested by reducing the temperature to 4°C for 6 hours prior to imaging at 37°C (Fig 7A). To synchronize growth of control hyphae with the cold-paused group, control hyphae were also initially arrested at 4°C for 6 hours and then grown continuously. Upon resuming growth, a 3-fold increase in branching was observed for growth-arrested hypha (Fig 7B), confirming that stalling tip extension in the absence of mechanical impedance is sufficient to induce branching at the hypha tip.

### Induction of hyphal branching can be phenocopied by chelation of iron

**Fig 8:**
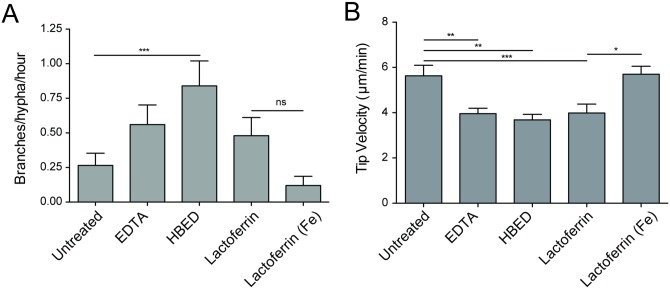
Apical branching is induced following chelation of iron. (A) Treatment with iron chelators increased branching compared to controls. EDTA provides non-specific chelation of free metal ion, while HBED and lactoferrin provide specific chelation of Fe. Lactoferrin (Fe) provides an iron-saturated negative control for lactoferrin iron chelation. (B) Iron chelation significantly reduced hyphal tip growth speed. Lack of growth inhibition by iron-saturated lactoferrin demonstrates the specificity of lactoferrin activity in this assay. N = 25 hyphae scored per condition from 3 experiments. Errors bars: mean ± SEM. Statistics: One-way ANOVA with Tukey’s post test. Adjusted p values: *p≤0.05, **p≤0.01, ***p≤0.001, ****p≤0.0001.

Neutrophil-derived lactoferrin has been shown to inhibit hyphal growth by chelation of iron [8]. To test whether iron chelation could induce branching, we exposed actively growing hyphae to non-specific (EDTA) and specific (hydroxybenzyl ethylenediamine—HBED, Lactoferrin) iron chelators (Fig 8). The addition of HBED resulted in a statistically significant increase in hyphal branching (Fig 8A), while treatment with EDTA or active Lactoferrin also significantly slowed hyphal tip growth and appeared to double average branching (from 0.26 to 0.56 and 0.48 branches/hypha/hour respectively, Fig 8). Importantly, Lactoferrin-induced growth inhibition was not observed for iron-saturated Lactoferrin (“Lactoferrin (Fe)”, Fig 8B), demonstrating that the growth inhibition effect of Lactoferrin treatment was dependent on its capacity to chelate iron.

### Branch induction slows hypha growth and increases vulnerability to attack by human neutrophils

**Fig 9:**
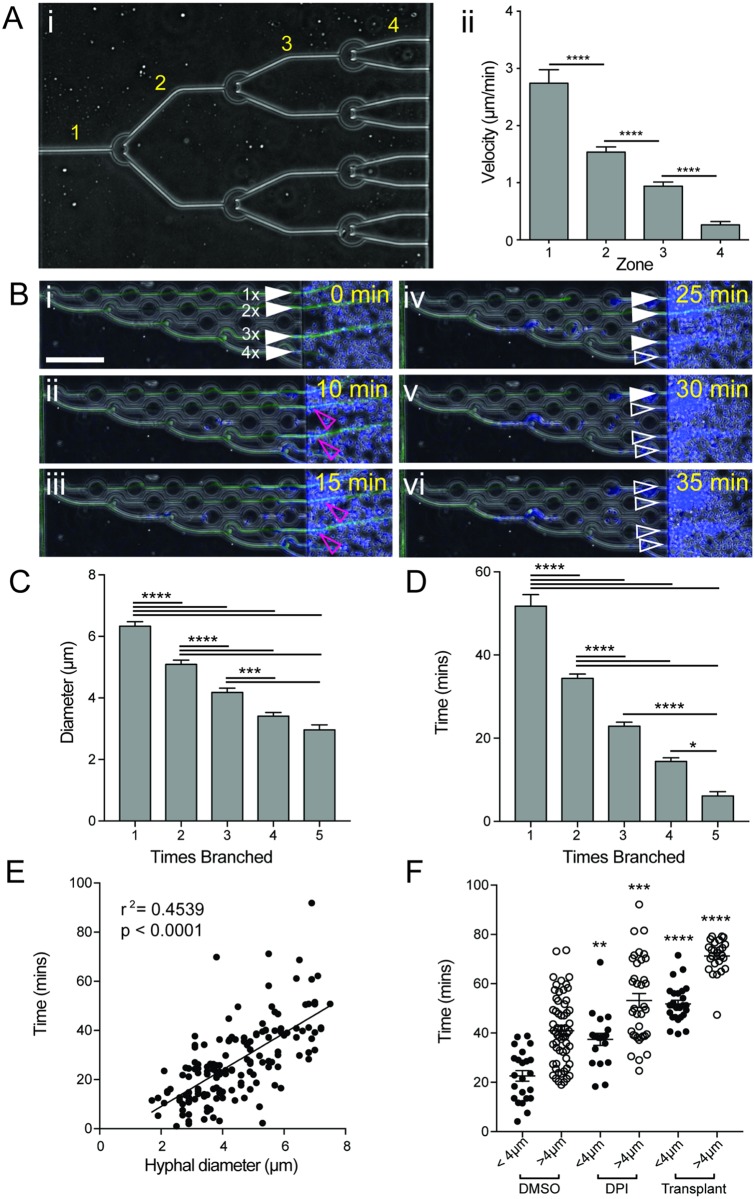
Branching slows growth and increases hyphal vulnerability. (A) Sequential branching was induced by crescent-shaped obstacles to allow comparison of hyphal growth after multiple branches. Hyphae were induced to branch 3 times, increasing the number of growing tips exponentially from 1 to 8 (i, S5 Movie). Comparison of hyphal tip velocity in zones 1–4 (i) demonstrated a significant reduction in hyphal growth speed with each branching event (ii). N ≥ 4 measurements for n ≥ 4 hyphae per zone. (B) Highly branched hyphae are more susceptible to killing by neutrophils. Staggered branch induction allowed comparison of hypha (full white arrowheads) that had undergone different numbers of branching events (i, S6 Movie). Hyphae that had been branched the most were killed first (iv, open white arrowhead), while more established hypha took longer to kill (v-vi). (C) Branching significantly reduces hyphal diameter. Hyphal diameter was measured for hyphae following compound branch induction. Reduced diameters were observed following branching, with a minimum diameter of 2 μm observed. N ≥ 11 hyphae measured per condition from ≥ 3 fields of view. (D) Thinner hyphae are more susceptible to neutrophil-mediated killing. (i) Bar graph shows that more highly branched hyphae are killed more quickly by neutrophils, as indicated by loss of hyphal cytoplasmic EGFP fluorescence. N ≥ 11 hyphae measured per condition from ≥ 3 fields of view. (E) Scatterplot shows time taken for neutrophils to kill hyphae correlates well with hyphal diameter, with thicker hyphae taking longer to kill. N = 157 hyphae scored. (F) Neutrophils with reduced ROS (DPI-treated) or isolated from at-risk patients (transplant recipients, S1 Table) take longer to kill both thick (>4μm diameter) and thin (<4 μm diameter) hyphae. N≥ 20 hyphae scored per group, neutrophils from N≥ 3 individuals sampled per group. Error bars: mean ± SEM. Statistics: One-way ANOVA with Tukey’s post test. Adjusted p values: *p≤0.05, **p≤0.01, ***p≤0.001, ****p≤0.0001.

The robust branching frequency induced by the novel crescent-shaped “tip-trap” obstacle (Fig 6C, S4 Movie) facilitated measurement of the effect of branch induction on hyphal growth speed. By comparing hyphae during induction of sequential branching, we observed that tip velocity was reduced post-branch, relative to the number of times branches were induced (Fig 9Ai and 9Aii, S5 Movie).

This approach also enabled us to test the relationship between the degree of hyphal branching and the susceptibility of hypha to destruction by neutrophils. Addition of neutrophils to the device following induction of sequential branching demonstrated that neutrophils were better able to compromise the cell wall integrity of highly branched hyphae, as indicated by reduced time to loss of hyphal cytoplasmic EGFP fluorescence (Fig 9B and 9D, S6 Movie), likely due to their smaller diameter (Fig 9C and 9E).

Importantly, neutrophils with reduced capacity to produce ROS (DPI treated) and neutrophils isolated from at-risk patients (kidney transplant recipients receiving immunosuppressive therapy, S1 Table) were less efficient at killing primary hyphae, exhibiting 42% and 59% killing respectively, compared to 86–87% in control groups. The time required for killing was also increased, even for thin, highly branched hyphae generated using sequential mechanical branch induction (Fig 9F). These observations support a model in which induction of branching may inhibit hyphal infection by slowing hyphal penetration, and increase hyphal vulnerability to destruction by healthy neutrophils, so long as sufficient numbers of active neutrophils are present. However, if neutrophil numbers are insufficient, or their activity suppressed, branch induction provides an evasive mechanism by which hyphae can avoid neutrophil interaction to continue, and in some cases amplify, hyphal growth.

## Discussion

Here we demonstrate that neutrophil-hypha interactions correlate with branching of *Aspergillus fumigatus* hyphae. The ability of neutrophils to pause hyphal tip extension is key to this fungal response. The response could be phenocopied by stalling of hyphal tip growth mechanically, thermally, and via chelation of iron.

Branching is associated with lower hyphal tip extension velocity, and results in thinner hyphae with increased surface area-to-volume ratios, although total fungal mass may not be affected. In the presence of excess neutrophils, thinner hyphae exhibit increased vulnerability to destruction. Mutations that induce constitutive hypha hyperbranching in *A*. *fumigatus* are associated with severe growth inhibition and decreased virulence in infection models [17–19], likely due to the slower speeds at which they are able to penetrate the tissue, and potentially an increased vulnerability to destruction. Induction of hyperbranching by plasma may provide a mechanism that sensitizes hypha to destruction by innate immune cells, but may also have negative implications for clot formation and blockage of vessels in infection [35–37]. Clot formation was robustly induced by hyphae in the presence of platelets and Fibrinogen, although formation of cross-linked Fibrin was dispensable for induction of branching. In the context of our iron chelation experiments, the ability of Fibrinogen alone to induce branching is likely linked to its activity as a iron-binding protein [38]. Identification of specific plasma factors that induce hyphal hyperbranching remains an interesting avenue for further research.

Our study expands upon the current understanding of the activity of neutrophils in aspergillosis. Neutrophils are assumed to be ineffective at killing phagocytosed conidia *in vitro* due to the tough outer coating [39] and because conidia appear to remain immunologically silent to neutrophils until they begin to germinate [40,41]. Observations are emerging suggesting that the primary role of neutrophils is to kill hyphae [42,43]. Working in concert with non-cellular immune components, such as platelets [33], defensins [44] and complement [45,46], extracellular neutrophil mechanisms act to restrict invasive growth and destroy hyphal structures.

This study has demonstrated that interaction with neutrophils induces loss of hyphal tip polarity and induction of *de novo* hyphal tip formation. Given that this effect could be phenocopied by chelation of iron, neutrophil-derived Lactoferrin is a good candidate to mediate this interaction. Neutrophil-derived Lactoferrin has previously been shown to inhibit conidial growth [8,47], but was not found to significantly affect hyphal viability [8]. However, hyphae exposed to human neutrophils show increased expression of ion-transporter catalysis mediators, including the 4-fold up-regulation of the ferric-chelate reductase Afu6g13750 [48]. Fungal ferric-chelate reductases are functionally homologous to components of the human NADPH oxidase complex [49], and are important for both iron acquisition and production of ROS. Given the central role of intracellular ROS in maintenance of hyphal tip polarity [20,21] and the role of ion sequestration in neutrophil suppression of fungal growth [7,50], the link between neutrophils, iron-sequestration, and loss of tip polarity is well supported.

Furthermore, localized extracellular ROS generated by neutrophils at sites of interaction along the length of the hyphae appeared to coincide with *de novo* tip formation in some instances (Fig 3C) suggesting a parallel pathway by which neutrophil interaction might induce localized re-polarization and lateral hyphal branch formation.

The current study was enabled by innovative microfluidic devices that allowed us to study interactions between neutrophils and fungal hyphae in unprecedented detail by: 1) Guiding the growth of individual hypha tips down parallel channels, allowing us to track hypha growth speed over defined distances; 2) Initiating interaction and branching with highly controlled temporal and spatial constrains; 3) Integration of imaging and biochemical assays; and 4) Providing a high-throughput system to trigger branching with utmost control over location, number, and timing of branching.

Although predominantly associated with diseases that suppress the immune system, opportunistic fungal infections are becoming an increasing problem in the clinic [51]. Modern medical treatments involving organ and bone marrow transplant rely heavily on immunosuppression of the patient to avoid tissue rejection and graft-versus-host disease. Although a direct effect of immunosuppressant therapy on neutrophil activity remains controversial, our previous studies indicate that neutrophils from these patients exhibit reduced activity against *A*. *fumigatus* [27]. Prophylactic treatment of immunosuppressed patients with broad-spectrum antibiotics, aimed at reducing risk of bacterial infection, is standard practice [52]. This not only encourages development of multidrug-resistant bacterial strains [53], but also affects commensal bacteria niches [54]. In combination with host immunosuppression, this scenario creates an environment permissive for opportunistic fungal infection [55–57].

Our studies indicate that, if insufficient neutrophil numbers are present, hyphal branching acts as an evasive maneuver, allowing the hyphae to continue penetrative growth past the interaction point. Given the susceptibility of neutropenic patients to fungal infection, these observations suggest that interacting with low neutrophil numbers may actually make the infection more aggressive by increasing the number of tissue-penetrating hyphal tips.

Although still relatively rare, rates of opportunistic fungal infection are steadily rising [51], and once established, infections are very difficult to treat [56]. Shared eukaryotic biology underpins the toxicity of many current antifungal medications to the host [58], while few attempts at development of new therapies have been made in the last decades [59].

The ability to modulate either neutrophil or hyphal behavior in the context of this interaction has direct relevance to the outcome of infection. Correction of neutrophil numbers or perhaps even stimulation of existing neutrophil functions in neutropenic patients is likely to improve outcome by increasing hyphae destruction. Similarly, blockage of *de novo* hyphal tip formation by targeting key fungal proteins such as WetA [60] may reduce hyphal virulence by reducing the number of hyphae for neutrophils to target. Conversely, induction of hyperbranching, such as can be achieved by targeting regulatory factors such as Calcineurin [61,62], may slow hyphal growth enough to allow destruction by neutrophils.

The assay we describe here may provide a high-throughput screening platform for identification of drugs affecting these important pathways. In particular, the novel “tip-trap” devices we present allow exceptional control over induction of hyphal branching, would be well suited to identification of compounds that inhibit *de novo* tip formation, and could be applied to a wide range of fungal species that exhibit hyphal growth.

## Materials and Methods

### Device design and fabrication

The microfluidic device was designed with three key elements, (1) the neutrophil loading ports and outer channel (Fig 1Ai, in purple), (2) the central conidia loading port and inner chambers (Fig 1Aii, in green) and (3) the neutrophil-hypha interaction channels (Fig 1Aiii). The twelve imaging fields on each device contain nine 10 x 675 μm interaction channels spaced 65 μm apart, each with seven 50 μm diameter swarming chambers spaced at 50 μm intervals.

Both the outer neutrophil loading channel and inner conidia port were fabricated at 50 μm depth, while the interaction channels at 10 μm depth to facilitate single-plane imaging.

The master wafer was fabricated using standard photolithographic technologies with Mylar photomasks (FineLine Imaging, Colorado Springs, CO). Polydimethylsiloxane (PDMS) (Sylgard 184, Elsworth Adhesives, Wilmington, MA) microfluidic devices were made by replica molding from the master wafer. Briefly, PDMS and curing agent were combined at a 10:1 ratio, mixed thoroughly, and poured over the master wafer. PDMS was then degassed for at least 30 min, then baked at 65°C for at least 3 hours. The PDMS was then peeled from the master wafer, and holes punched. The two outer neutrophil loading holes were punched with a 0.75 mm puncher, while the inner conidia loading port was punched using a 1.2 mm puncher. Each device was then cut out using an 8 mm puncher (Harris Uni-Core, Ted Pella Inc., Redding, CA). Following oxygen plasma treatment, devices and 12-well glass-bottom plates (MatTek Corp. Ashland, MA) were bonded at 85°C on a hotplate for 10 mins, and allowed to cool slowly.

### Preparation of devices

One day prior to the experiment, devices were primed with Iscove’s Modified Dulbeco’s Medium (IMDM) with 20% fetal bovine serum (FBS) by loading the device with a gel-loading tip, inner port first followed by outer ports, such that media formed a dome on top of the device covering all three loading ports. Devices were subjected to vacuum for 10 mins, then allowed to re-pressurize for at least 20 mins, or until all air bubbles within the device were gone. The entire device was then submerged in media (2 mL per well in 12 well plate), and 0.5 μL of conidia (at 5 x 10^7^ conidia/mL) loaded into the central port using a gel-loading tip, with care taken not to pipette into the surrounding media. Loaded devices were then incubated overnight, allowing germination of conidia and growth of hyphae into the outer chambers and interaction channels.

Once hyphae began to enter the interaction channels, neutrophils were delivered through the loading port and into the outer channels.

For N-formyl-Met-Leu-Phe (fMLP) experiments, fMLP (Sigma) at 100nM was delivered through the central loading port.

### Immune cell preparation and treatments

Immune cells were isolated from human peripheral blood samples from healthy donors aged 21 years and older (Research Blood Components, LLC Allston, MA) drawn into 10 mL sodium heparin vacuum tubes (Vacutainer, Becton Dickinson). Nucleated cells were isolated by HetaSep gradient, then immune subsets were separated using EasySep kits from STEMCELL Technologies (Vancouver, Canada). Neutrophils were isolated using a Human Neutrophil Enrichment Kit, eosinophils with a Human Eosinophil Enrichment Kit, monocytes using a Human Monocyte Enrichment Kit, and lymphocytes using a Human Lymphocyte Enrichment Kit.

Human macrophages were differentiated from monocytes using standard techniques. Briefly: Monocytes were suspended in 1640 Roswell Park Memorial Institute medium (RPMI) and incubated for 2 hours at 37°C with 5% CO_2_ to allow monocyte adherence. After 2 hours, the non-adherent population was removed, and the remaining cells washed with ice cold Phosphate Buffered Saline (PBS) and trypsinized for 5 min at 37°C. Cells were then suspended in 1640 RPMI + 10% FBS medium containing Granulocyte macrophage colony-stimulating factor (GM-CSF), and incubated at 37°C with 5% CO_2_ for 24 hours. The GM-CSF medium was then replaced with 1640 RPMI with 1% Pen/Strep and 10% human serum (Sigma). Cells were maintained in 1640 RPMI with Pen/Strep and 10% human serum for 7 days with the medium changed every 3 days.

Prior to loading into the device, cells were typically stained with Hoechst 33342 (Life Technologies, Carlsbad, CA) according to the manufacturers protocol and re-suspended at 6 x 10^7^ cells/mL in media. For imaging ROS, CellROX DeepRed reagent (ThermoFisher) was added at 12.5 μM to both to the media within the device and used to pre-stain neutrophils for 30 min prior to co-delivery with cells.

For all drug treatments, neutrophils were pre-treated for at least 30 mins with drug (diphenyleneiodonium chloride (DPI—Sigma) at 200 μM, 4-aminobenzoic hydrazide (ABAH—Sigma) at 500 μM, N-Methoxysuccinyl-Ala-Pro-Val-chloromethyl ketone (NEi—ThermoFisher) at 100 μM) and washed prior to delivery.

### *A*. *fumigatus* preparation

*A*. *fumigatus* conidia of strain 293 expressing cytosolic EGFP of RFP were a kind gift from Jatin Vyas, prepared by Nida Khan using standard methods. Upon receipt, conidia were filtered using spin columns with 5 μm pores (Thermo Scientific) to isolate un-germinated spores and re-suspended at 5 x 10^7^ conidia/mL in dH_2_O.

### Plasma preparation and delivery

To prepare plasma fractions, including WP, PRP, and PPP, blood was collected from healthy volunteers in accordance with IRB protocols (2009-P-000295). Platelets were prepared from blood collected into 2.9 mL trisodium citrate Vacutainer tubes (Sarstedt, Nümbrecht, Germany). PRP was prepared by centrifugation of the whole blood at 210 g, 22°C, for 20 minutes. The PRP supernatant was gently pipetted into two tubes. In one tube, 20% volume acid citrate dextrose solution (ACD) (Boston Bioproducts Inc., Ashland, MA) was added and the PRP was incubated at 37°C until the start of the experiment. The second tube was centrifuged at 1900 g, 22°C, for 10 minutes. The PPP was removed, leaving a platelet pellet. IMDM with 20% FBS was used to re-suspend the platelet pellet, and ACD was added to the PPP and the platelet suspension. Platelets were stained with Calcein (Thermo Fisher Scientific Inc, Grand Island, NY). HT-plasma was prepared by heating PPP to 56°C for 30 mins. All plasma preparations were kept at 37°C until the start of the experiment.

### Iron chelation

Iron chelation was achieved by delivering chelating treatments throughout the entire device, via the central port. EDTA (Sigma-Aldrich) was delivered at 10 μM, HBED (Santa Cruz Biotechnology) at 100 μM, and Lactoferrin groups at 100 μM. Human Lactoferrin derived from human breast milk was purchased from Sigma in both active and inactive (iron-saturated) forms.

### Microscopy and analysis

All time-lapse imaging experiments were performed at 37°C with 5% carbon dioxide on a fully-automated Nikon TiE microscope, using a Plan Fluor 10x Ph1 DLL (NA = 0.3) lens. Image capture was performed using NIS-elements (Nikon Inc, Melville, NY) and analysis performed using FIJI (FIJI is just ImageJ, NIH) and Python scripts. NIS-elements and FIJI software were used to normalize the background color, and convert the file to an AVI. Python scripts used OpenCV (opencv.org) and TrackPy (http://soft-matter.github.io/trackpy/v0.3.0/) toolkits. OpenCV was used for background subtraction, automated cell counts and NET identification. A 5-frame rolling background filter was applied to remove cells that remained stationary for more than 10 mins from the tracking analysis. Tracks were based on relative velocity and position and informed by the inherent physical limitations imposed by the microfluidic channels. The TrackPy toolkit was used for cell tracking using the cells positions identified previously. Automated cell identification was based on cell size and shape, NET production was based on color and size, and hyphal tip velocity was measured by manually tracking tips in FIJI.

### Statistics

Graphing and statistical analysis was performed using Prism (v6.0c, GraphPad Software Inc. La Jolla, CA). Statistical analysis included two-tailed Student’s t-tests for comparing pairs of normally distributed data, and one-way ANOVA with Tukey’s post *hoc test* for comparing datasets containing multiple treatment groups.

### Ethics statement

Samples from kidney transplant patients (S1 Table) were collected at the Massachusetts General Hospital (MGH) with informed consent and IRB approval under protocol number 2009-P-002826.

## Supporting Information

S1 Table Transplant patient information. (PDF)

S1 Fig Hyphal growth and neutrophil recruitment. (A) Hyphae show a small but significant bias toward growing down channels located at the opposite end of the outer chambers rather than those at the side.(B) Neutrophil recruitment numbers for different experimental conditions. Note the lower recruitment observed in DPI-treated groups compared to the higher recruitment observed for devices primed with fMLP.Error bars: Box and whisker plots show 10–90% confidence intervals. Statistics: Student’s T-test.
(TIF)

S1 Movie Time-lapse movie of interactions between growing *Aspergillus fumigatus* hyphae and human neutrophils. *A*. *fumigatus* (green, GFP) grow through the interaction channels and interact with human neutrophils (blue, Hoechst), which actively migrate from the outer channel towards the hyphae.
(AVI)

S2 Movie Time-lapse movie of neutrophil-induced hyphal branching. Movie shows three examples of neutrophil-induced hyphal branching induced by increasing numbers of neutrophils. More neutrophils are better able to slow hyphal growth and attack freshly formed branches, but one hypha almost always escapes interaction to continue growing.
(AVI)

S3 Movie Induction of hyphal hyperbranching correlates temporally with formation of cross-linked fibrin clots. Movie shows time-lapse of hyphae growing into a chamber filled with human platelet-rich plasma. Induction of a hyperbranching phenotype appears to correlate with formation of cross-linked fibrin within the chamber.
(AVI)

S4 Movie Induction of hyphal branching by mechanical impedance of tip extension. Movie shows hyphae (green, GFP) growing through channels that contain crescent-shaped “tip-trap” obstacles. Upon interaction with these obstacles, hyphal branching is induced in a robust manner that decouples branching from neutrophil interaction.
(AVI)

S5 Movie Sequential branch induction by mechanical impedance. Movie shows hypha growing through a device that incorporates multiple “tip-trap” obstacles for compounded induction of hyphal branching. Induction of multiple branches reduces both the speed of hyphal extension, and the diameter of sequentially branched hyphae.
(AVI)

S6 Movie Killing of branched hyphae by neutrophils. Movie shows time-lapse of neutrophils interacting with hyphae after they have been induced to branch. This device is designed to allow comparison of hyphae that have only branched once (top channel) with hyphae that have branched multiple times (bottom three channels). Neutrophils are able to kill thinner, more highly branched hyphae faster, as measured by quenching of hyphal cytoplasmic GFP fluorescence upon neutrophil interaction.
(AVI)
