# Supplementary material for: Neutrophil Interactions Stimulate Evasive Hyphal Branching by Aspergillus fumigatus
Source: PLoS Pathog. 2017 Jan 11;13(1):e1006154. doi: 10.1371/journal.ppat.1006154 (PMC5261818; doi:10.1371/journal.ppat.1006154)
Supplement: S1 Table — (PDF) [file ppat.1006154.s001.pdf]

Table S1. Transplant patient information

Patient	Organ	Biopsy Result	Time since transplant (days)	Treatment (Daily doses)
1	Kidney (2nd)	CNI toxicity	16	MMF: 500 mg<br>Prednisone: 15 mg<br>TAC: 2 mg
2	Kidney	ACR Banff 2A	50	MMF: 1g<br>Belatacept: 650 mg<br>Prednisone: 20 mg
3	Kidney	Chronic acute cellular rejection	3039	MMF: 500 mg<br>Prednisone: 5 mg<br>TAC: 6 mg
