# Supplementary figures and images for: Neutrophil Interactions Stimulate Evasive Hyphal Branching by Aspergillus fumigatus
Source: PLoS Pathog. 2017 Jan 11;13(1):e1006154. doi: 10.1371/journal.ppat.1006154 (PMC5261818; doi:10.1371/journal.ppat.1006154)

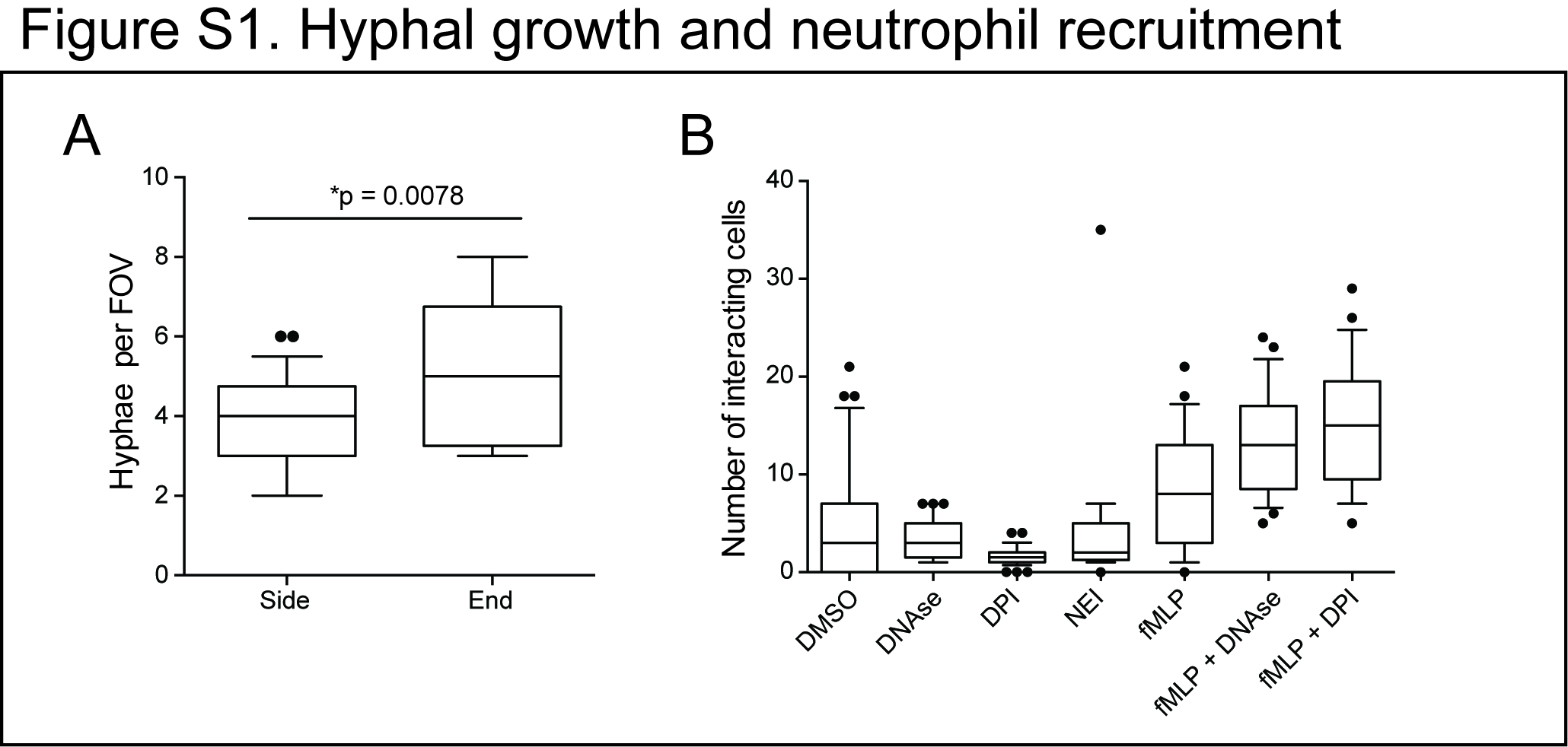

Supplement: S1 Fig — (A) Hyphae show a small but significant bias toward growing down channels located at the opposite end of the outer chambers rather than those at the side.(B) Neutrophil recruitment numbers for different experimental conditions. Note the lower recruitment observed in DPI-treated groups compared to the higher recruitment observed for devices primed with fMLP.Error bars: Box and whisker plots show 10–90% confidence intervals. Statistics: Student’s T-test. (TIF) [file ppat.1006154.s002.tif]
